# Review of roles of RNA-binding proteins on NAFLD and the related pharmaceutical measures

**DOI:** 10.17305/bb.2025.12465

**Published:** 2025-05-15

**Authors:** Changjin Li, Fan Yang, Zuohui Yuan, Xiaoguo Wei

**Affiliations:** 1 Department of Gastroenterology, Gansu Provincial Hospital, Lanzhou 730000, Gansu, China

**Keywords:** RNA-binding proteins, RBP, nonalcoholic fatty liver disease, NAFLD, therapy, mechanism

## Abstract

Nonalcoholic fatty liver disease (NAFLD) is the most common chronic liver disease worldwide and poses a serious threat to public health. NAFLD is considered a risk factor for metabolic syndrome (MS) and is closely associated with type 2 diabetes mellitus (T2DM), obesity, dyslipidemia, and cardiovascular disease. Recently, increasing attention has been paid to the role of RNA-binding proteins (RBPs) in the pathogenesis of NAFLD. A growing body of research has linked RBPs—such as human antigen R (HuR), sequestosome 1 (p62), polypyrimidine tract-binding protein 1 (PTBP1), and heterogeneous nuclear ribonucleoproteins (hnRNPs)—to lipogenesis and inflammation, both of which contribute to NAFLD through mechanisms involving transcriptional regulation, alternative splicing, RNA stability, polyadenylation, and subcellular localization. However, these findings are often fragmented and lack a comprehensive synthesis. The interactions and mechanisms between RBPs and NAFLD have not yet been thoroughly reviewed. This article provides an overview of the roles and mechanisms of various RBPs in NAFLD, summarizing current knowledge with the aid of figures and tables. In particular, it highlights the influence of HuR on NAFLD through multiple pathways, categorizing its effects based on increased or decreased expression. Furthermore, it reviews drugs that alleviate NAFLD by modulating RBPs, aiming to offer valuable insights for drug-targeted therapies based on RBP regulatory networks.

## Introduction

Nonalcoholic fatty liver disease (NAFLD) is one of the most prevalent liver disorders worldwide, affecting over 25% of the adult population and imposing significant social and economic burdens [1]. It is characterized by the accumulation of lipids in hepatocytes and elevated levels of liver-related enzymes. Steatosis is diagnosed when at least 5% of liver cells exhibit excessive fat deposition, particularly in individuals who either abstain from alcohol or limit their intake to no more than 20 g/day for women and 30 g/day for men [2, 3]. NAFLD encompasses a spectrum ranging from simple intrahepatic fat accumulation (steatosis) to nonalcoholic steatohepatitis (NASH), which involves hepatocyte inflammation and cell death and may progress to fibrosis, cirrhosis, and hepatocellular carcinoma (HCC) [4, 5]. NASH plays a central role in the progression of NAFLD, with hepatic fibrosis identified as the primary predictor of liver-related mortality [6]. A recent model predicts a 178% increase in liver-related deaths associated with NASH by 2030 [7]. More recently, researchers in the field have proposed the term “MAFLD” (metabolic associated fatty liver disease) as a more appropriate overarching label, reflecting the growing recognition of the condition’s metabolic underpinnings. Like NAFLD, MAFLD represents the hepatic manifestation of a multisystem disorder [8]. Nevertheless, this review will continue to use the term NAFLD to align with its widespread usage in the literature reviewed.

The leading explanation for the etiology and pathophysiology of NAFLD is the “multiple factors” hypothesis. This model encompasses fat accumulation, insulin resistance (IR), oxidative stress, mitochondrial dysfunction, endoplasmic reticulum stress (ERS), disruptions in hepatic lipid and bile acid (BA) metabolism, alterations in the gut microbiota, and genetic predispositions [9]. NAFLD is a multifactorial disease strongly associated with metabolic syndrome (MS), type 2 diabetes mellitus (T2DM), and obesity, with IR recognized as a central component in its pathogenesis [10]. As the disease progresses, IR worsens and activates lipogenic enzymes via sterol regulatory element-binding protein 1c (SREBP1c), thereby promoting lipogenesis [11, 12]. Furthermore, IR diminishes insulin’s ability to suppress lipolysis in adipose tissue, leading to elevated levels of free fatty acids (FFAs) delivered to the liver [13, 14].

RNA-binding proteins (RBPs) play a central role in coordinating RNA processing and post-transcriptional gene regulation (PTGR), including the maturation, localization, stabilization, and translation of both coding and non-coding RNAs [15]. RBPs interact with RNA through RNA-binding domains (RBDs), which are primarily found in the 5′ and 3′ untranslated regions (UTRs) of target RNAs. Most RBPs contain multiple RBDs, enhancing their affinity and specificity for target mRNAs. A single RBP can regulate the expression of numerous mRNAs, while multiple RBPs may bind to the same mRNA, either cooperating or competing in their regulatory functions [16, 17]. Loss of RBP function or functional mutations can disrupt cellular homeostasis and contribute to metabolic disorders such as NAFLD [18, 19].

Although existing studies have explored the role of RBPs in NAFLD, their scope is often limited by the vast diversity of RBPs. The primary aim of this work is to provide a comprehensive review of RBPs that have a relevant impact on NAFLD, with a detailed summary of their roles and underlying mechanisms. Additionally, growing interest in drug development targeting RBP-regulated pathways has led to the emergence of several RBP-based therapeutic strategies. This review also discusses such drug candidates, reflecting the critical role of RBPs and their RNA interactions in the progression of NAFLD.

## RBPs in NAFLD

### Hu proteins

The embryonic lethal abnormal vision (ELAV)/Hu proteins represent an important family of RBPs, comprising four mammalian members encoded by distinct genes: HuB, HuC, HuD, and human antigen R (HuR) (also known as human antigens B, C, D, and R). These proteins play key roles in enhancing gene expression at the post-transcriptional level [20]. HuR, which is broadly expressed across tissues, stabilizes and/or promotes the translation of mRNAs encoding pro-inflammatory mediators in the cytoplasm, serving as a critical regulator of inflammatory and immune responses [21]. In contrast, HuB, HuC, and HuD are primarily expressed in neurons, where they contribute to neuronal differentiation, axonal growth, and the maintenance of neuronal integrity [22].

### HuR

**Figure 1.**
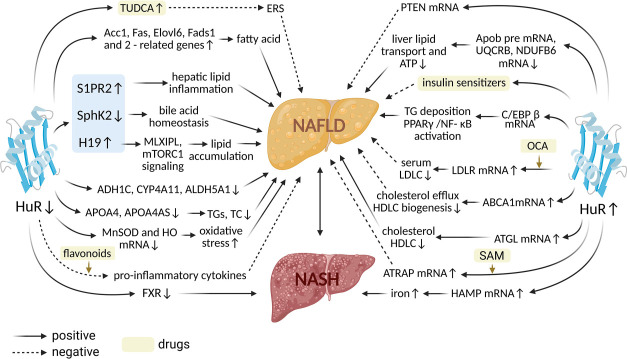
**Interactions between HuR and according RNAs in NAFLD.** Modulating the expression of HuR, through either upregulation or downregulation, can influence the expression of related mRNAs and substances, as well as the efficacy of specific pharmaceuticals. This modulation can have both beneficial and detrimental effects on the onset and progression of NAFLD. In this context, solid arrows denote positive effects, while dashed arrows indicate negative effects. HuR: Human antigen R; NAFLD: Nonalcoholic fatty liver disease; TG: Triglyceride; TC: Total cholesterol; SAM: S-adenosylmethionine; LDLR: Low-density lipoprotein receptor; ATRAP: AT1R-associated protein; ATGL: Adipose triglyceride TG lipase; NASH: Nonalcoholic steatohepatitis; C/EBPβ: CCAAT/enhancer-binding protein beta; PPARγ: Proliferator-activated receptor gamma; NF-κB: Nuclear factor kappa B; FXR: Farnesoid X receptor; APOA4: Apolipoprotein A-IV; MnSOD: Manganese-dependent superoxide dismutase; TUDCA: Tauroursodeoxycholic acid; mTORC1: Mechanistic target of rapamycin complex 1; Acc1: Acetyl-CoA carboxylase; Fas: Fatty acid synthase; Elovl6: Elongation of very-long-chain fatty acids member 6; Fads1&2: Fatty acid desaturases 1 and 2; S1PR2: Sphingosine-1-phosphate receptor 2; SphK2: Sphingosine kinase 2; ERS: Endoplasmic reticulum stress.

HuR, encoded by the ELAV-like 1 (Elavl1) gene, is located on human chromosome 19p13.2 [23]. Its mRNA is approximately 6 kb in length and encodes a protein with a molecular weight of 36 kDa. Functionally, HuR regulates post-transcriptional gene expression by modulating the stability and activity of its target RNAs. Structurally, HuR contains three RNA recognition motifs (RRMs): a tandem RRM1 and RRM2, connected by a flexible linker, and a C-terminal RRM3 [24]. Although primarily localized in the nucleus, HuR can translocate to the cytoplasm upon specific stimuli, a process mediated by a shuttling sequence located between RRM2 and RRM3 [20]. In the cytoplasm, HuR interacts with its target mRNAs to exert diverse regulatory effects—most commonly promoting mRNA stability and enhancing translation, although in certain tissues it may also suppress these functions [25, 26]. Recently, HuR has gained attention for its critical roles in cell signaling, inflammation, fibrosis, and the development of HCC [27]. Several studies have also identified hepatic HuR as a key player in NAFLD progression through its regulation of lipid and glucose metabolism, modulation of lipid transport, and suppression of adipogenesis [28–30]. A recent study reported a significant reduction in HuR expression in the livers of mice fed a high-fat diet (HFD). However, in contrast to this observation, a separate study by Zhang et al. [31] found that hepatic HuR deficiency actually worsened HFD-induced NAFLD. These conflicting results may be due to differences in the HFD conditions used across studies involving HuR-deficient mice. In the latter study, HuR deficiency led to increased levels of triglyceride (TG) and cholesterol ester (CE) species, along with decreased expression of genes involved in cholesterol biosynthesis and the BA-activated farnesoid X receptor (FXR)/retinoid X receptor (RXR) pathway. As a result, these mice exhibited heightened inflammation and fibrosis, ultimately progressing to HCC-like tumor development [28]. In HuR-deficient livers, serum taurocholic acid (TCA), macrophage markers, innate immune response genes, multiple chemokines, and the long non-coding RNA (lncRNA) LINC01018 were significantly upregulated. In contrast, levels of tauroursodeoxycholic acid (TUDCA)—a BA known to inhibit ERS—were reduced [28, 32]. Additionally, genes involved in fatty acid biosynthesis, including acetyl-CoA carboxylase (Acc1), fatty acid synthase (Fas), elongation of very-long-chain fatty acids member 6 (Elovl6), and fatty acid desaturases 1 and 2 (Fads1 and Fads2), were markedly upregulated [32]. As summarized in Figure 1, HuR plays a regulatory role in NAFLD progression by modulating ERS and fatty acid metabolism.

The lncRNA H19, a 2.3 kb RNA molecule, has emerged as a key regulator of hepatic lipid metabolism and ERS [33, 34]. H19 promotes steatosis and lipid accumulation by modulating several pathways, including the miR-130a/peroxisome proliferator-activated receptor gamma (PPARγ) axis, MLX-interacting protein-like (MLXIPL) expression, and mechanistic target of rapamycin complex 1 (mTORC1) signaling [35, 36]. Mechanistically, HuR deficiency leads to upregulation of H19 and sphingosine-1-phosphate receptor 2 (S1PR2), while suppressing sphingosine kinase 2 (SphK2) expression, thereby promoting inflammation and hepatic lipid accumulation (as illustrated in Figure 1). In summary, HuR functions as a critical regulator of hepatic lipid metabolism, enterohepatic BA homeostasis, inflammation, and fibrosis. Hepatocyte-specific deletion of HuR exacerbates these pathological changes, underscoring its potential as a therapeutic target for NAFLD [32].

Phosphatase and tensin homolog deleted on chromosome 10 (PTEN) functions as a lipid phosphatase that dephosphorylates phosphatidylinositol-3,4,5-trisphosphate (PIP3), thereby inhibiting downstream signaling of phosphatidylinositol 3-kinase (PI3K) and reducing AKT activity [37]. In the liver, PTEN plays a critical role in regulating lipogenesis, glucose metabolism, and hepatocyte homeostasis. Liver-specific PTEN deficiency promotes NAFLD and hepatocarcinogenesis, while also improving glucose tolerance [38]. In contrast, mice overexpressing PTEN are protected against hepatic steatosis [39] (see Figure 1). HuR binds to the 3′UTR of PTEN mRNA, enhancing its stability and translation. In HuR-deficient mice, overexpression of PTEN alleviates diet-induced hepatic fat accumulation, highlighting a protective axis involving HuR and PTEN in NAFLD pathogenesis [30].

HuR interacts with intron 24 of Apob pre-mRNA, the 3′UTR of UQCRB mRNA, and the 5′UTR of NDUFB6 mRNA, thereby regulating Apob mRNA splicing and the translation of UQCRB and NDUFB6. Hepatocyte-specific deletion of HuR reduces the expression of Apob, UQCRB, and NDUFB6 in mice, impairing ATP synthesis and hepatic lipid transport, which in turn exacerbates HFD-induced NAFLD [31]. As illustrated in Figure 1, HuR deficiency worsens NAFLD under HFD conditions. Given HuR’s complex role in insulin sensitivity, a therapeutic strategy combining HuR modulation with insulin sensitizers—such as thiazolidinediones, glucagon-like peptide-1 (GLP-1) receptor agonists, biguanides (e.g., metformin), and dipeptidyl peptidase IV (DPP-4) inhibitors—may provide more effective treatment for NAFLD [40]. Interestingly, increases in the expression of HuR, NDUFB6, CYCS, Apob-100, UQCRB, and Apob-48 following oral metformin administration may represent an off-target effect of metformin.

LINC01018 is a non-conserved intergenic lncRNA located on chromosome 5 and is highly expressed in the liver. In patients with NAFLD, hepatic expression of LINC01018 is significantly reduced compared to healthy controls. Interestingly, dietary intervention with low carbohydrate intake in NAFLD patients restores LINC01018 expression, along with the upregulation of genes associated with its activity. LINC01018 modulates fatty acid metabolism through its interaction with HuR [41]. In humanized mouse models, reducing hepatic HuR expression by more than 60% results in a marked decrease in the expression of three key genes regulated by LINC01018: ADH1C, CYP4A11, and ALDH5A1. This downregulation contributes to the development and progression of NAFLD (Figure 1).

Apolipoprotein A-IV (APOA4) is a plasma lipoprotein primarily synthesized in the liver and small intestine [1]. In mice, APOA4 promotes hepatic TG secretion, thereby increasing plasma TG levels [42]. APOA4-AS, an antisense lncRNA, acts as a coordinated regulator of APOA4 expression, exhibiting a parallel expression pattern. Both APOA4-AS and APOA4 are elevated in the livers of individuals with NAFLD. Knockdown of APOA4-AS *in vitro* and *in vivo* reduces APOA4 expression, leading to decreased plasma TG and total cholesterol (TC) levels in mice. Mechanistically, APOA4-AS exerts its regulatory function by directly binding to HuR, which stabilizes APOA4 mRNA. Deletion of HuR leads to downregulation of both APOA4-AS and APOA4 transcripts [43]. As illustrated in Figure 1, HuR contributes to increased TG and TC levels through its stabilizing effect on APOA4-AS and APOA4.

CCAAT/enhancer-binding protein beta (C/EBPβ), a member of the C/EBP transcription factor family, plays a pivotal role in initiating adipogenesis and contributes to diabetes pathogenesis by regulating key metabolic enzymes such as phosphoenolpyruvate carboxykinase (PEPCK) in both adipose tissue and the liver. In models of NASH induced by a methionine–choline-deficient (MCD) diet, C/EBPβ expression is markedly upregulated. Hepatic deletion of C/EBPβ protects against excessive TG accumulation, inflammation, ERS, and oxidative stress. In contrast, overexpression of C/EBPβ exacerbates activation of PPARγ and nuclear factor kappa B (NF-κB) signaling pathways [44]. Previous studies have shown that HuR facilitates the nucleocytoplasmic transport and stability of C/EBPβ mRNA during the early stages of adipogenesis [45, 46] (see Figure 1). While direct evidence in the context of liver disease is still lacking, these findings suggest that HuR may regulate C/EBPβ expression and thereby influence the development and progression of NAFLD.

HAMP, the gene encoding human hepcidin, is primarily expressed in the liver and plays a central role in regulating iron absorption from the duodenum and iron release from macrophages [47]. In the context of NAFLD, studies have shown that elevated levels of saturated fatty acids in the liver enhance HuR translocation from the nucleus to the cytoplasm. This translocation promotes HuR binding to the 3′UTR of HAMP mRNA, resulting in increased HAMP expression in hepatocytes [48]. Modulating this HuR-mediated regulation of HAMP may offer therapeutic potential in slowing or preventing the progression of NAFLD (Figure 1).

Treatment with obeticholic acid (OCA) increases the expression of low-density lipoprotein receptor (LDLR) in the liver, leading to reduced plasma levels of low-density lipoprotein cholesterol (LDL-C) in mice. This effect is mediated through activation of the FXR, which induces the expression of HuR—a post-transcriptional regulator that stabilizes LDLR mRNA [49]. Deletion of HuR blocks OCA-induced upregulation of LDLR and impairs plasma cholesterol clearance via the LDLR pathway (Figure 1).

ABCA1 plays a key role in reverse cholesterol transport by facilitating cholesterol efflux and the biogenesis of high-density lipoprotein (HDL). HuR enhances ABCA1 expression by binding to its 3′UTR and stabilizing its mRNA. Silencing HuR reduces ABCA1 expression and impairs cholesterol efflux to apolipoprotein A1 (APOA1) in both hepatocytes and macrophages [50]. As illustrated in Figure 1, HuR supports reverse cholesterol transport and HDL formation by promoting ABCA1 expression.

A recent study found that mice lacking HuR specifically in adipose tissue were more susceptible to HFD-induced metabolic dysfunction, exhibiting increased IR and inflammation. HuR reduces lipid accumulation and lowers serum TC and HDL cholesterol (HDL-C) by stabilizing and promoting the translation of adipose TG lipase (ATGL) mRNA [29] (Figure 1).

HuR protects liver cells from oxidative damage caused by excessive fat accumulation by regulating the stability of manganese-dependent superoxide dismutase (MnSOD) and heme oxygenase-1 (HO-1) mRNAs. Researchers have identified ten HuR-binding sites within the 3′UTR of MnSOD mRNA, and HuR knockdown leads to reduced MnSOD mRNA and protein levels [51]. In mice, decreased MnSOD levels intensify oxidative stress, worsening the pathological features of NASH [52]. Similarly, lower MnSOD levels have been observed in NASH patients, with certain genetic variants affecting MnSOD function and correlating with increased NASH risk [53, 54]. Recent studies have also shown that administration of the HO-1 inducer hemin significantly alleviates steatosis, inflammation, and fibrosis in mice fed an MCD diet, while also reducing serum ALT and AST levels by inhibiting both canonical and non-canonical Wnt signaling pathways [55]. In MCD-induced models of NASH, HuR has been shown to regulate the stability of both MnSOD and HO-1 mRNAs. Reduced HuR expression correlates with decreased MnSOD and HO-1 levels, suggesting that impaired HuR-mediated regulation of antioxidant enzymes may contribute to the progression from simple steatosis to NASH [56] (Figure 1).

Angiotensin II (Ang II) and its type 1 receptor (AT1R) have been identified as key contributors to the progression of NAFLD. AT1R blockers have shown promise in ameliorating fatty liver and are being explored as potential therapeutic agents for NAFLD [57, 58]. AT1R-associated protein (ATRAP, also known as AGTRAP) functions as a negative regulator of AT1R by directly binding to it, thereby attenuating the effects of Ang II [59]. S-adenosylmethionine (SAM), a methyl donor involved in epigenetic and protein modifications, has been shown to influence the expression of ATRAP by modulating HuR activity [60]. Specifically, ATRAP mRNA interacts with HuR, and SAM preserves HuR methylation, which is essential for the nucleocytoplasmic shuttling of ATRAP mRNA. This, in turn, enhances ATRAP protein production and may help mitigate NAFLD progression (Figure 1). These findings underscore HuR’s critical role in mRNA transport, a regulatory mechanism with implications for NAFLD pathogenesis [61]. Additionally, several bioactive compounds—including berberine, quercetin, and apigenin—have been found to inhibit HuR, thereby reducing the expression of HuR-regulated genes, particularly those involved in inflammation. This anti-inflammatory effect may be beneficial in the treatment of NAFLD (Figure 1) [62].

Circular RNA poly(A) binding protein nuclear 1 (circPABPN1) has been shown to recruit HuR, thereby preventing its interaction with PABPN1 mRNA and ultimately reducing PABPN1 translation [63]. However, the potential role of PABPN1 in the onset and progression of NAFLD remains largely unexplored [64, 65]. Based on the above analysis, we summarize the key interactions between HuR and various molecular pathways implicated in NAFLD pathogenesis (Figure 1).

### Human antigen D

HuD plays a critical role in regulating TG levels in pancreatic β-cells. Research has shown that reduced HuD expression leads to increased intracellular TG accumulation in βTC6 cells by downregulating the post-transcriptional expression of insulin-induced gene 1 (INSIG1), a key inhibitor of lipid synthesis. This downregulation also enhances the nuclear localization of SREBP1c, thereby activating genes involved in lipogenesis [66]. However, similar regulatory effects of HuD have not yet been observed in the liver, highlighting the need for further investigation into its potential role in hepatic lipid metabolism.

### lncRNAs

lncRNAs are single-stranded RNA molecules ranging in length from approximately 200 base pairs to 10 kilobases and represent the most abundant class of non-coding RNAs in the human genome—outnumbering microRNAs (miRNAs) [67, 68]. LncRNAs regulate nearly every aspect of post-transcriptional RNA processing, including pre-mRNA splicing, cleavage and polyadenylation, translational control, nuclear export, RNA stability, localization, and editing [17]. RBPs can recognize and bind specific RNA sequences to form heterogeneous nuclear ribonucleoprotein (hnRNP) complexes. In mammalian cells, the hnRNP family comprises at least 20 nuclear RBPs [69, 70]. Several hnRNPs associated with lncRNAs—such as hnRNPI, hnRNPU, and hnRNPA1—have been implicated in NAFLD. However, there remains a substantial gap in our understanding of their precise roles, molecular mechanisms, and expression profiles in the context of NAFLD.

### hnRNPI/Polypyrimidine tract-binding protein 1(PTBP1)

PTBP1, also known as PTB or hnRNP I, belongs to the PTBP family, which also includes PTBP2 and PTBP3. All three proteins share a common structure comprising four RRMs [71]. PTBP1 is widely expressed in non-neural tissues and in neurogenic cells. PTBP2 (also known as nPTB) is predominantly expressed in neurons, spermatocytes, and myoblasts, while PTBP3 (also referred to as ROD1) is primarily found in hematopoietic and hepatic tissues. PTBP3 functions as a splicing factor and has been implicated in HCC development [72]. Its downregulation inhibits activation of the PI3K/AKT signaling pathway, thereby suppressing HCC growth, migration, and invasion [73]. However, to date, no studies have directly linked PTBP3 to NAFLD. PTBP1, originally characterized as a histone-associated hnRNP in HeLa cells, binds to hnRNA and is also referred to as hnRNP I [74, 75]. It plays essential roles in mRNA stabilization, alternative splicing, and nucleocytoplasmic transport by binding to polypyrimidine-rich tracts in pre-mRNAs [75]. The lncRNA H19 shares multiple binding sites with PTBP1 [76]. Through its interaction with PTBP1, H19 modulates hepatic metabolism and contributes to the progression of fatty liver disease [77]. Interestingly, overexpression of H19 has also been reported to protect against obesity and improve insulin sensitivity, according to research by Schmidt et al. [78]. Conversely, inhibition of H19 enhances the differentiation of human adipose-derived stem cells and promotes lipid accumulation by targeting PTBP1 [76]. Disruption of hepatic lipid homeostasis induces the expression of both H19 and PTBP1, which enhances their interaction. This promotes PTBP1 binding to Srebp1c mRNA and protein, resulting in greater mRNA stability, increased proteolytic cleavage, enhanced nuclear translocation, and elevated transcriptional activity of SREBP1c—further stimulating lipogenesis. These findings reveal an H19/PTBP1/SREBP1 feedforward loop that amplifies signaling and contributes to NAFLD progression [77]. In addition, human lncRNA metabolic regulator 1 (hLMR1) enhances PTBP1 binding to the promoters of Sc5d, Lss, Fdps, and hydroxymethylglutaryl-CoA synthase 1 (Hmgcs1), thereby promoting the transcription of genes involved in cholesterol metabolism [79]. Circular RNA (circRNA) circMBOAT2, located on chromosome 2p25, promotes lipid metabolic reprogramming in intrahepatic cholangiocarcinoma (ICC) through the circMBOAT2/PTBP1/FASN axis. It binds to PTBP1 and protects it from ubiquitin-dependent degradation. This altered lipid profile influences cell membrane composition, energy metabolism, and redox balance, potentially affecting NAFLD progression [80]. Furthermore, hyperglycemia and elevated FFA levels stimulate pancreatic and duodenal homeobox 1 (PDX1) expression, which in turn enhances the transcription of metastasis-associated lung adenocarcinoma transcript 1 (MALAT1). This may contribute to β-cell dysfunction via the PDX1/MALAT1/PTBP1 axis, potentially linking this pathway to NAFLD [81]. Finally, PTBP1 also affects the invasiveness and metastatic potential of hepatocarcinoma by regulating the alternative splicing of Axl exon 10 [82].

### hnRNPU

A recent study identified a novel lncRNA, regulator of hyperlipidemia (lncRHL), which suppresses hepatic very-low-density lipoprotein (VLDL) secretion. lncRHL exerts its effect by binding to and stabilizing hnRNP U (hnRNPU). In turn, hnRNPU transcriptionally activates Bmal1, leading to reduced VLDL secretion. When lncRHL is deficient, hnRNPU becomes destabilized and degraded more rapidly, resulting in suppressed Bmal1 transcription and increased VLDL secretion in liver cells. The lncRHL/hnRNPU/Bmal1/microsomal TG transfer protein (MTTP) axis presents a promising regulatory pathway for maintaining hepatic and plasma lipid homeostasis [83]. Inactivation of hnRNPU in liver cells exacerbates HFD-induced NASH by inducing a truncated form of the tyrosine kinase receptor B (TrkB), which promotes liver inflammation, hepatocyte death, and fibrosis [84]. Additionally, the family with sequence similarity 3D (FAM3D)–formyl peptide receptor 1 (FPR1) signaling axis has been shown to upregulate hnRNPU expression. This increase enhances lipid oxidation and reduces fat accumulation in obese mice by recruiting the glucocorticoid receptor (GR) to the promoter region of the short-chain acyl-CoA dehydrogenase (SCAD) gene [85].

Brown fat lncRNA 1 (Blnc1) and lnc-BATE1 are both key regulators of brown adipocyte differentiation [86]. lnc-BATE1 binds to hnRNPU in trans, directing lncRNAs to specific subnuclear domains to facilitate their functional activity [87]. This interaction forms a ribonucleoprotein complex that is essential for brown adipogenesis [86]. The transcription factor zinc finger and BTB domain-containing 7b (Zbtb7b) plays a crucial role in the development of brown and beige adipose tissue by promoting the assembly of the Blnc1/hnRNPU complex [88].

Lnc-RAP-1, also known as Firre, is another lncRNA that binds to hnRNPU [87]. Inhibition of lnc-RAP-1 has been shown to impair lipid accumulation and reduce the expression of adipocyte markers during white preadipocyte differentiation [89]. Additionally, hnRNPU recruits Blnc1 to the transcription factor EBF2, forming the Blnc1/hnRNPU/EBF2 ribonucleoprotein complex, which promotes the expression of thermogenic genes [90]. While these mechanisms are clearly involved in fat metabolism, their roles in liver metabolism and NAFLD remain largely unexplored.

### hnRNPA1

**Figure 2.**
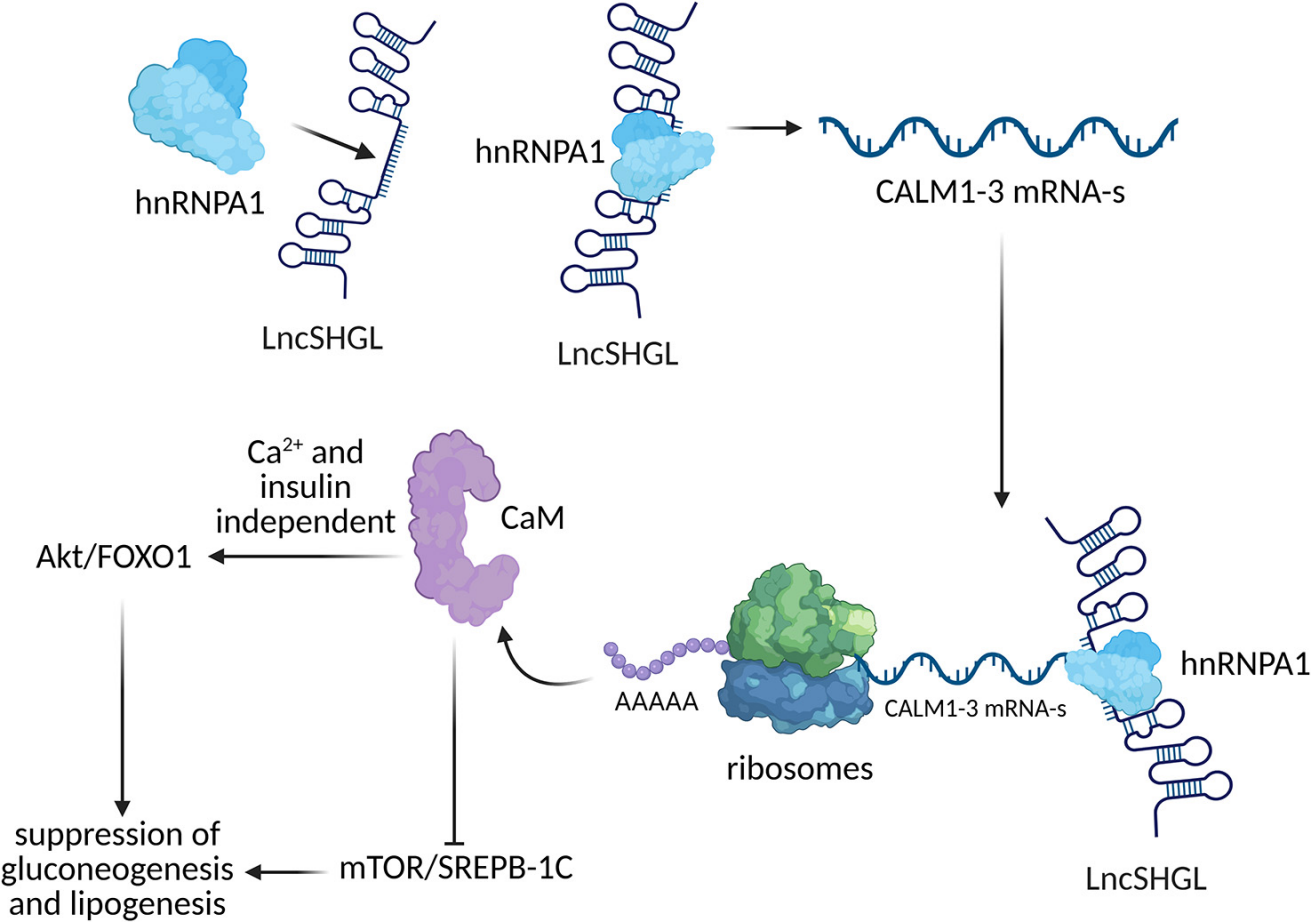
**Proposed mechanism of lncSHGL in inhibiting liver gluconeogenesis and lipogenesis.** The proposed mechanism by which LncSHGL inhibits hepatic gluconeogenesis and lipogenesis involves the recruitment of hnRNPA1, which enhances the translational efficiency of CALM1-3 mRNAs, leading to an increase in intracellular CaM protein levels. Elevated CaM protein activates the Akt signaling pathway while concurrently repressing the mTOR/SREBP-1C pathway. This interplay ultimately suppresses gluconeogenesis and lipogenesis in hepatocytes. Adapted from Wang et al. Long noncoding RNA lncSHGL recruits hnRNPA1 to suppress hepatic gluconeogenesis and lipogenesis. Diabetes 2018;67(4):581–93, with permission from the publisher [95]. hnRNPA1: Heterogeneous nuclear ribonucleoprotein A1; CaM: Calmodulin; SREBP1c: Sterol regulatory element-binding protein 1c; FoxO1: Forkhead box protein O1; mTOR: Mammalian target of rapamycin.

hnRNPA1, a highly abundant member of the hnRNP family, is known to stabilize mRNAs and regulate their expression [70]. Recent findings by Zhao et al. [91] revealed significantly elevated levels of TGs and TC in both liver tissues and serum of hnRNPA1-knockout mice. Loss of hnRNPA1 in murine skeletal muscle was shown to exacerbate IR and hepatic steatosis under HFD conditions. This effect is attributed to hnRNPA1’s interaction with glycogen synthase 1 (gys1) mRNA, which promotes glycogen synthesis and helps maintain insulin sensitivity. Additionally, Gui et al. [92] demonstrated that hnRNPA1 regulates lipid metabolism by binding to the lncRNA H19 and enhancing the translation of key fatty acid oxidation genes, such as carnitine palmitoyltransferase 1B (CPT1b) and PPARγ coactivator 1-alpha (PGC1α), thereby improving IR. In a steatosis model using HepG2 cells supplemented with FFAs, hnRNPA1 binding to the 5′UTR of SREBP-1a was shown to increase via activation of the p38 MAPK signaling pathway [93]. This interaction promotes cap-independent translation of SREBP-1a, activating SREBP-responsive genes involved in lipid metabolism. Furthermore, the lncRNA SHGL suppresses both lipogenesis and gluconeogenesis in the liver by recruiting hnRNPA1 to enhance translation of calmodulin (CALM1–3) mRNAs. This results in increased cellular calmodulin (CaM) protein levels, which activate the PI3K/Akt signaling pathway and inhibit the mTOR/SREBP-1c axis [94]. These combined effects independently suppress gluconeogenesis and lipogenesis in hepatocytes, offering a promising therapeutic strategy for treating hepatic steatosis via the lncSHGL/hnRNPA1 regulatory axis (Figure 2) [95].

### hnRNPA2B1

Lnc-HC forms a ribonucleoprotein complex with hnRNPA2B1, targeting transcripts of CYP7A1 and ABCA1, two key genes involved in lipid and cholesterol metabolism [96]. *In vitro* studies have shown that this interaction leads to the nuclear retention and subsequent degradation of CYP7A1 and ABCA1 mRNAs, resulting in cholesterol accumulation within hepatocytes [97].

### hnRNPC

Umbilical cord-derived mesenchymal stem cell extracellular vesicles (UCMSC-EVs) deliver circ-Tulp4 into hepatocytes, where circ-Tulp4 inhibits the HNRNPC/ABHD6 axis. This inhibition reduces apoptosis and alleviates DM-NAFLD, offering a novel therapeutic strategy for targeting DM-NAFLD through modulation of cell death pathways [98].

### p62/insulin like growth factor 2 (IGF2) mRNA binding protein 2

The human IGF2 mRBPs (IMP1–3 or IGF2BP1–3) were first identified in 1999 due to their interaction with IGF2 leader 3 mRNA [99]. These proteins play a key role in modulating RNA dynamics in a transcript-specific manner across the genome [100]. While the expression of Imp1 and Imp3 markedly declines after birth, Imp2 remains widely expressed postnatally [101]. p62, a splice variant of IGF2BP2 lacking exon 10, retains all six characteristic RNA-binding motifs despite this deletion [102].

Studies have shown that liver-specific overexpression of p62 induces histological steatosis in approximately 60% of animals, without causing overt liver damage [103]. Transgenic animals with elevated p62 levels on a normal diet develop fatty liver [103] and are also prone to developing NASH [104]. When these p62-overexpressing animals are fed an MCD diet, they exhibit increased expression of Ccl2 [104]. In contrast, IMP2-deficient mice are highly resistant to HFD-induced fatty liver [105]. These mice display reduced fat mass—especially during HFD feeding—along with lower circulating lipid levels, decreased hepatic TG accumulation, and improved glucose tolerance and insulin sensitivity [105]. Notably, most lipid species are elevated in p62-induced steatosis, with TGs showing the most significant increase [106]. When p62 liver-specific transgenic mice are fed an MCD diet, they develop earlier and more severe fibrosis [104, 107], suggesting that IMP2 accelerates NAFLD progression. Furthermore, following treatment with diethylnitrosamine (DEN), livers of p62-transgenic mice exhibit increased inflammation and an enhanced ductular reaction (DR), characterized by dedifferentiated cells that drive the progression toward steatohepatitis-associated cirrhosis [108].

Overexpression of IMP2 in hepatocytes may disrupt miRNA regulation, impairing the translation efficiency of its target RNAs and leading to aberrant fatty acid metabolism, thereby contributing to steatosis [109]. Animal studies suggest that p62 promotes NASH progression by driving hepatic iron deposition and free cholesterol production, which in turn lead to lipid peroxidation and inflammation via NF-κB activation [110]. Research by Stephan Laggai on p62 transgenic mice revealed an elevated C18:C16 fatty acid ratio and increased expression of fatty acid elongase 6 (ELOVL6), accompanied by liver inflammation [111]. ELOVL6 expression is specifically associated with steatotic processes and has been linked to hepatic inflammation [112]. Mechanistically, p62 promotes hepatic C18 fatty acid production through SREBP1-dependent induction of ELOVL6, contributing to NASH in both mice and humans [113]. ELOVL6 overexpression has been associated with NASH development. In mice fed a HFD, miR-130b expression increases [114], while activation of the AKT pathway suppresses hepatic adipogenesis and gluconeogenesis in NAFLD models [115]. Recent findings indicate that in NAFLD mice, downregulation of miR-130b-5p reduces lipid accumulation by upregulating IGFBP2, whereas miR-130b-5p overexpression enhances lipid accumulation by inhibiting IGFBP2. In HFD-fed mice, downregulation of miR-130b-5p or overexpression of IGFBP2 boosts IGFBP2 levels and increases AKT phosphorylation, resulting in the suppression of lipid synthesis genes (SREBP-1, SCD1, LXRα, ChREBP, Acc1, and Fas). This ultimately inhibits lipid accumulation and improves IR in NAFLD. In summary, miR-130b-5p exacerbates lipid accumulation and IR in NAFLD by inhibiting the AKT pathway via suppression of IGFBP2 [116]. Hepatocyte-specific deletion of IMP2 modestly promotes diet-induced fatty liver by impairing fatty acid oxidation, due to increased degradation of IMP2 client mRNAs, such as Cpt1a and Pparα, without significantly affecting lipogenic gene expression [117]. Repression of IGFBP2 is common in both NAFLD and NASH patients and is often attributed to DNA methylation, with expression levels varying across NAFLD cases [118]. Notably, hypermethylation of IGFBP2 precedes the onset of hepatic steatosis in dietary NAFLD models—even when mice are metabolically stable—highlighting its potential as an early biomarker for liver disease risk. Additionally, IGFBP2 expression is age-dependent, showing decreased levels in young mice prone to HFD-induced obesity [119].

### Tristetraprolin (TTP)

TTP contains two zinc finger motifs that enable RNA binding. Although its role in liver physiology remains incompletely understood and somewhat inconsistent across studies, evidence suggests that hepatic TTP contributes to the progression of steatosis, inflammation, and fibrosis. Deletion of TTP prevents steatosis in mice fed an MCD diet, possibly by enhancing VLDL secretion [120]. Conversely, bone marrow-specific deletion of TTP reduces serum levels of TGs, TC, and VLDL/LDL, but promotes hepatic steatosis and alters the expression of genes involved in lipid metabolism and inflammation, such as Srebp1, Saa1, and Ccr2 [121]. TTP shares common mRNA targets with HuR, another RBP implicated in hepatic steatosis. Therefore, downregulation of TTP may indirectly promote steatosis by reducing competition with HuR for these shared targets [122].

**Figure 3.**
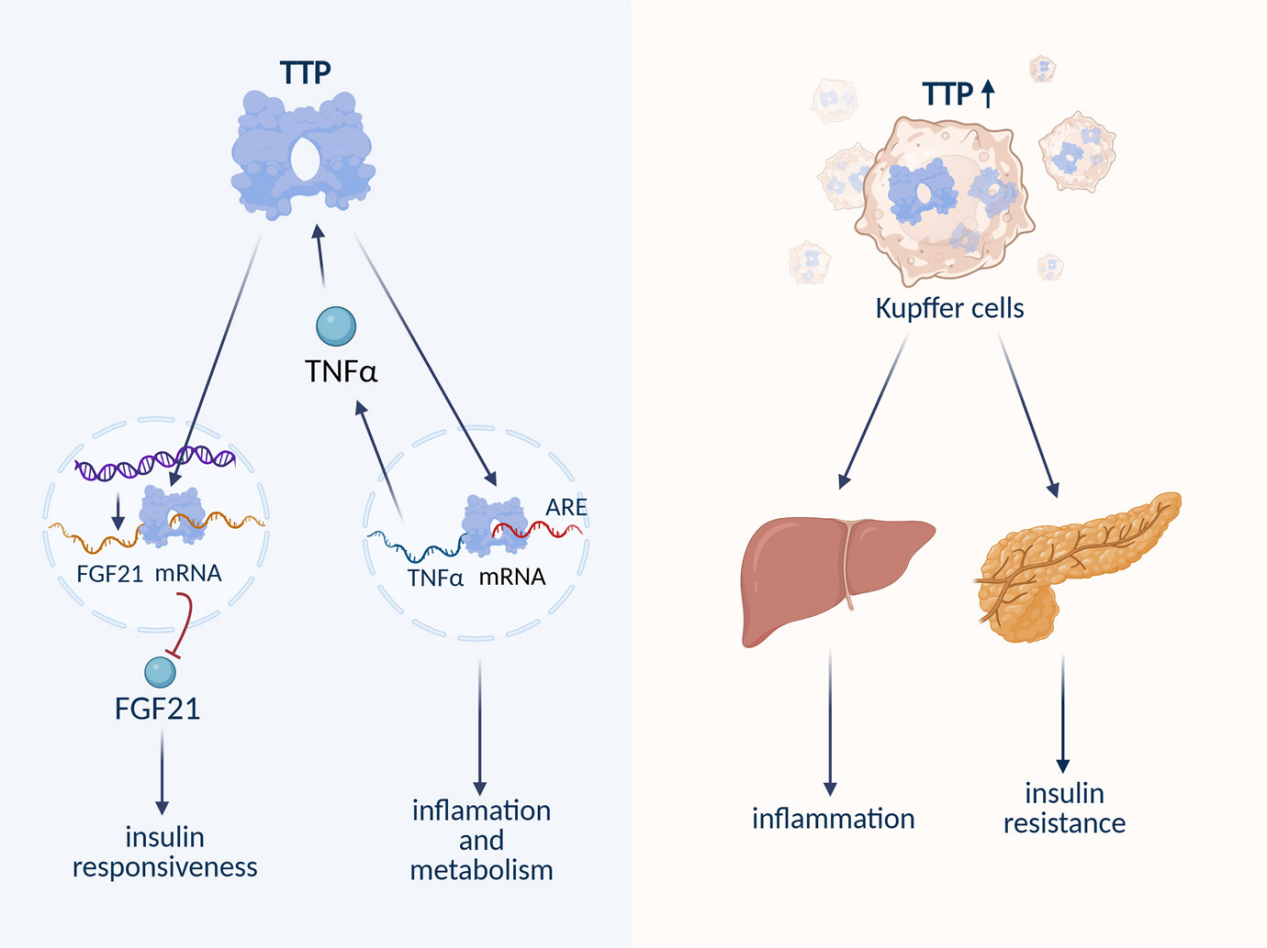
**RNA-TTP interaction in IR and hepatic fat deposition.** TTP controls TNF-α levels by binding to its mRNA’s ARE region, influenced by TNF-α, affecting inflammation and metabolism. It also suppresses FGF21 mRNA, impacting insulin sensitivity. Elevated TTP in KCs worsens liver inflammation and IR. TTP: Tristetraprolin; IR: Insulin resistance; KC: Kupffer cell; ARE: AU-rich element; FGF21: Fibroblast growth factor 21.

Carbon monoxide (CO) promotes the sequestration of plasminogen activator inhibitor-1 (PAI-1) into stress granules (SGs), and CO-induced activation of TTP enhances PAI-1 degradation during SG assembly. This CO-dependent TTP activation reduces PAI-1 levels in SGs, potentially alleviating age-related NAFLD and highlighting TTP as a novel therapeutic target in age-associated liver disease [123]. Inhibition of linc-SCRG1 reduces the expression of fibrosis-related genes by suppressing TTP expression [124]. Linc-SCRG1 inhibits TTP, leading to the inactivation of hepatic stellate cell (HSC) phenotypes [124]. Studies have shown that metformin-induced activation of TTP decreases TNF-α production in Kupffer cells (KCs), thereby preventing hepatocyte necroptosis. Additionally, TTP-mediated destabilization of Ras homolog enriched in brain (Rheb) enhances lipophagy in primary hepatocytes and mouse liver, positioning TTP as a promising target for reducing hepatosteatosis [125]. TTP regulates TNF-α levels by binding to AU-rich elements (AREs) in the TNF-α mRNA transcript [126]. Interestingly, TNF-α itself can upregulate TTP expression, suggesting a reciprocal regulatory loop between TTP and TNF-α that may influence NAFLD progression [127]. Recent findings propose a novel role for TTP in metabolic regulation, particularly in hepatic glucose and lipid metabolism [128]. Sawicki et al. demonstrated that TTP post-transcriptionally represses fibroblast growth factor 21 (FGF21), a liver-derived hormone involved in insulin sensitivity. Loss of TTP results in elevated FGF21 levels, suggesting that hepatic TTP influences both liver and systemic insulin responsiveness. Thus, targeting hepatic TTP could represent a promising approach for treating NAFLD (Figure 3) [129].

### Cytoplasmic polyadenylation element-binding protein 1 (CPEB1)

**Figure 4.**
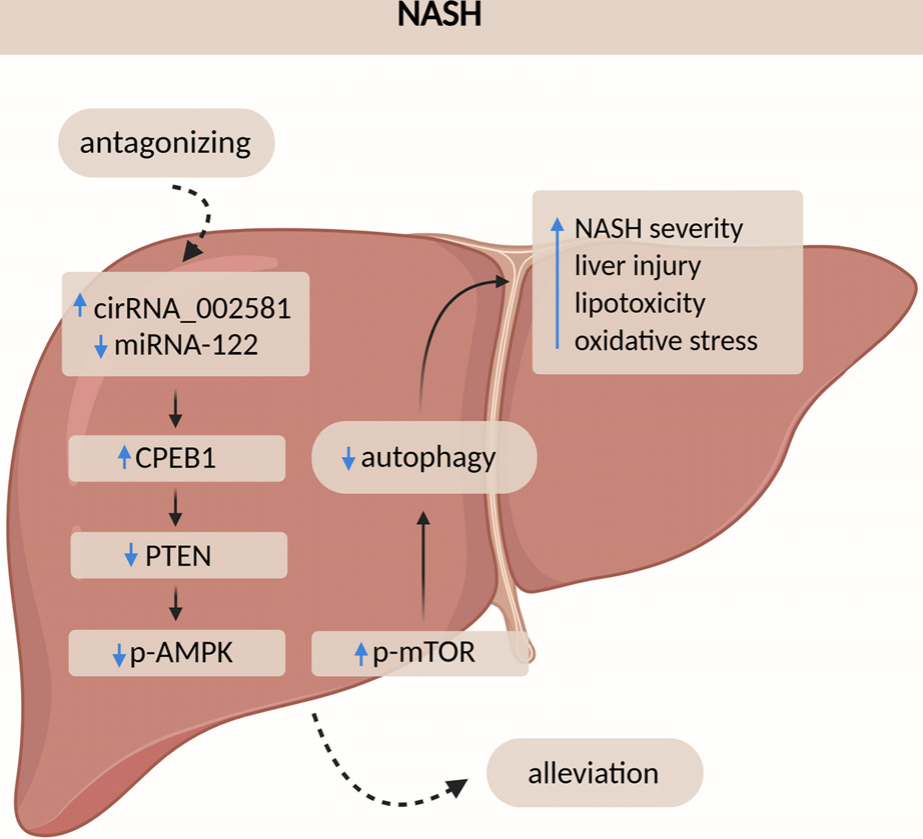
**A proposed model showing how antagonizing CircRNA_002581 alleviates NASH progression.** In NASH, CircRNA_002581 binds miR-122, boosting CPEB1 expression and impairing autophagy via the PTEN–AMPK–mTOR pathway, worsening NASH. Inhibiting CircRNA_002581 decreases miR-122 sequestration, reducing CPEB1 levels and partly restoring autophagy through PTEN–AMPK–mTOR, easing NASH. Adapted from Jin et al. Antagonizing circRNA_002581-miR-122-CPEB1 axis alleviates NASH through restoring PTEN-AMPK-mTOR pathway regulated autophagy. Cell Death Dis 2020;11(2):123, with permission from the publisher [135]. CircRNA: Circular RNA; NASH: Nonalcoholic steatohepatitis; CPEB1: Cytoplasmic polyadenylation element-binding protein 1; miR-122: MicroRNA-122; mTOR: Mammalian target of rapamycin.

CPEB1 is an mRNA-binding protein that regulates translation through cytoplasmic polyadenylation. It binds to the cytoplasmic polyadenylation element (CPE) in the 3′UTR of target mRNAs and interacts with three regulatory proteins: Gld2, PARN, and Maskin [130]. Elevated levels of CPEB1 have been implicated in pathological angiogenesis in chronic liver disease [131] and in promoting HCC stemness and chemoresistance [132]. In contrast, its homolog CPEB4 has been found to counteract hepatic steatosis under ERS conditions [133]. A previous study indirectly linked circRNA-002581 to CPEB1 regulation via sequestration of miRNA-122 (miR-122) [134]. More recently, the circRNA-002581–miR-122–CPEB1 axis has been shown to play an active role in NASH pathogenesis through modulation of the PTEN–AMPK–mTOR pathway and suppression of autophagy. In NASH models, knockdown of circRNA-002581 reduced lipid accumulation, ALT and AST levels, hydrogen peroxide (H_2_O_2_), pro-inflammatory cytokines, and apoptosis, while increasing ATP levels—suggesting circRNA-002581 as a potential therapeutic target for NASH (Figure 4) [135]. Furthermore, microarray analysis of mRNAs regulated by CPEB1 revealed that CPEB1 deficiency results in widespread impairment of insulin signaling. CPEB1 knockout mice exhibit IR, as CPEB1 normally represses the translation of Stat3 and Pten mRNAs. These findings suggest that CPEB1 may contribute to NAFLD by disrupting glucose homeostasis [136].

### Tat-activating regulatory DNA-binding protein-43 (TDP-43)

TDP-43 contains two RRM domains flanked by an N-terminal domain and a glycine-rich C-terminal region. Structurally, it resembles members of the hnRNP family and is involved in RNA processing [137]. Mutations in TDP-43 can cause its mislocalization from the nucleus to the cytoplasm, where it may aggregate abnormally [138]. Overexpression of TDP-43 has been shown to increase interleukin-6 (IL-6) levels in pre-adipocytes, macrophages, and adipocytes [139]. Steatosis activates NF-κB signaling via upstream activation of IKKβ, leading to increased production of TNF-α, IL-6, and IL-1β. These cytokines recruit and activate KCs, mediating inflammation and contributing to the progression of NASH [139]. A positive correlation has been observed between IL-6 levels and body mass index (BMI), and increasing soluble IL-6 receptor alpha (sIL-6Rα) and gp130/sIL-6Rβ levels has been shown to alleviate NAFLD in obesity [140]. Additionally, inhibiting IL-6/signal transducer and activator of transcription 3 (STAT3) signaling has been found to reduce I148M variant-mediated susceptibility to NAFLD [141]. TDP-43 also plays a role in lipid metabolism regulation through its interaction with the liver-specific lncRNA lncLSTR. This complex regulates Cyp8b1, a key enzyme in BA synthesis, enhancing the BA pool. This, in turn, promotes ApoC2 expression via FXR activation, leading to the stimulation of lipoprotein lipase and increased plasma TG clearance [142].

### Yes-associated protein 1 (YAP)

YAP1 or YAP plays a key role in liver repair, cell fate determination, and tumorigenesis. The lncRNA lncARSR prevents YAP phosphorylation, leading to activation of insulin receptor substrate 2 (IRS2) and increased expression of the SREBP-1c gene. IRS2 subsequently activates the PI3K/AKT pathway, inducing lipogenic gene expression and accelerating NAFLD progression [143]. Another study has shown that large tumor suppressor kinase 2 (LATS2) regulates YAP activity in NAFLD by modulating its phosphorylation status [144]. In macrophages, the STING–YAP axis regulates steatosis by reprogramming lipid metabolism through a pathway involving transmembrane protein 205 (TMEM205), mitofusin 2 (MFN2), and protein disulfide isomerase (PDI). TMEM205, a YAP target gene, activates AMPKα, which interacts with hepatocyte MFN2, promoting PDI–hypoxia-inducible factor-1α (HIF-1α) signaling and degradation of perilipin 2 (PLIN2) on lipid droplets (LDs). Macrophage-specific STING deficiency enhances nuclear YAP activity, reducing lipid accumulation and PLIN2 expression under HFD-induced oxidative stress [145]. Studies have also highlighted the involvement of the Wnt/β-catenin signaling pathway in regulating hepatic lipid metabolism [146, 147]. Ma et al. observed elevated levels of Y-box binding protein 1 (YB-1) and β-catenin in the livers of NAFLD mice. They further demonstrated that YB-1 influences lipid synthesis and β-oxidation via the Wnt/β-catenin pathway [148]. In addition, the lncRNA MAYA (MST1/2-Antagonizing for YAP Activation) promotes hepatocyte senescence by downregulating YAP expression [149], and hepatocyte senescence is known to contribute to the pathogenesis of NAFLD [150].

In monkeys with hepatic steatosis, increased nuclear localization of YAP has been observed in hepatocytes [151]. Studies have shown that Si-Ni-San, a traditional Chinese medicine formula, reduces YAP expression and mitigates lipid droplet accumulation in liver cells affected by NAFLD [152]. Rosmarinic acid (RA) also downregulates YAP protein levels and ameliorates NAFLD by modulating the YAP/TAZ–PPARγ/proliferator-activated receptor γ coactivator-1α (PGC-1α) signaling pathway [153]. Curcumol, a natural compound, inhibits hepatocyte senescence through YAP/nuclear receptor coactivator 4 (NCOA4)-mediated regulation of ferritinophagy in NAFLD. Supplementation with curcumol improves liver damage and reduces hepatic steatosis in HFD-fed golden hamsters [154]. The lncRNA SRD5A3-AS1 inhibits miR-1205, resulting in upregulation of NF2 expression. In turn, NF2 suppresses YAP activity, reducing cell proliferation and lowering the levels of inflammatory and fibrotic markers, such as IL-6, TGF-β1, and α-SMA in NAFLD [155]. Lian-Mei-Yin (LMY), a traditional Chinese medicine long used for treating liver disorders, has been found to reduce hepatic steatosis in both zebrafish and mouse models of NAFLD in a time- and dose-dependent manner. Its mechanism involves inhibition of Yap1-mediated activation of Foxm1, a key factor in NAFLD progression [156].

YAP expression in hepatic HSCs and KCs is critical for the development of fibrosis and the progression of NASH [157]. In mice with diet-induced NAFLD, hepatic expression of CYR61 increases in a YAP-dependent manner and is associated with fibrosis development [158]. As a key effector of the Hippo pathway, YAP plays an early and essential role in HSC activation [159], thereby promoting liver scarring during NASH progression [160]. Inhibition of the Hippo/YAP signaling pathway is necessary for magnesium isoglycyrrhizinate to suppress HSC inflammation and activation [161]. Recent research has identified a novel link between the gene CSN6, which stabilizes HMGCS1, and YAP activation through mevalonate metabolism. Targeting the CSN6–HMGCS1–YAP1 axis may reveal a potential therapeutic vulnerability in NAFLD-associated HCC [162]. Additionally, YAP activation in NASH may be linked to the DR. During NASH development, YAP activation occurs prior to DR and may contribute to it by promoting hepatocyte dedifferentiation [163].

### YB-1

YB-1 or YBX1 is a multifunctional DNA- and RBP characterized by a conserved cold shock domain (CSD) [164]. Its broad range of functions stems from its ability to interact with nucleic acids, form homomultimers, and assemble into complexes with other proteins. YBX1 regulates numerous DNA- and RNA-dependent processes, including transcription, splicing, translation, DNA repair, and mRNA stability [157, 165].

Adipocyte autophagy plays a significant role in the pathogenesis of NAFLD. Autophagy is elevated in the white adipose tissue (WAT) of mice fed a HFD, and suppression of autophagy in WAT has been shown to alleviate hepatic steatosis, inflammation, and fibrosis [166]. Studies have demonstrated that YB-1 facilitates adipogenesis by enhancing autophagy mediated by Unc-51–like kinases 1 and 2 (ULK1 and ULK2). Further investigation revealed that YB1 specifically binds to m^5^C-modified Ulk1 transcripts, stabilizing their mRNA. In addition to acting as an RBP, YB1 also functions as a DNA-binding protein that promotes Ulk2 transcription. Together, these actions increase ULK1 and ULK2 levels, thereby enhancing autophagy and promoting adipogenesis. Elevated YB1 expression in WAT increases autophagy and stimulates adipose tissue expansion in mice. Due to its regulatory roles in autophagy and adipogenesis, YB1 may represent a promising therapeutic target for combating obesity and related metabolic disorders [167]. The well-characterized lncRNA HOX Transcript Antisense RNA (HOTAIR) has also been shown to interact with YBX1, promoting cell proliferation through regulation of YBX1 target genes [168]. However, its direct impact on NAFLD remains to be fully elucidated and warrants further investigation.

### Eukaryotic initiation factor 4E (EIF4E)

EIF4E is an mRNA cap-binding protein essential for the interaction between mRNA and ribosomes, facilitating cap-dependent translation through its interaction with eukaryotic initiation factor 4G (eIF4G) [169]. Yan et al. [170] found that elevated plasma levels of EIF4E may causally contribute to the development of NAFLD in the European population. Wang et al. [171] showed that inflammatory stress enhances the phosphorylation of both mammalian target of rapamycin (mTOR) and EIF4E, which in turn promotes the translation of recombinant Cluster of Differentiation 36 (CD36). CD36 facilitates the uptake of long-chain fatty acids, leading to lipid accumulation and the onset of NAFLD [172]. Rapamycin has been shown to reduce CD36 expression by inhibiting the mTOR pathway and downstream phosphorylation events, thereby alleviating NAFLD [173]. Furthermore, a clinical trial conducted by Kubrusly et al. [174] demonstrated that EIF4E levels are elevated in patients with NASH-related cirrhosis.

### Astrocyte elevated gene-1 (AEG-1)

AEG-1, also known as metadherin (MTDH), is a 582-amino acid protein anchored to the endoplasmic reticulum membrane [175]. Elevated AEG-1 expression has been observed in individuals with NASH who developed steatosis following a HFD. Molecular analyses reveal that AEG-1 regulates fatty acid β-oxidation (FAO) by inhibiting the activation of PPAR alpha (PPARα), while simultaneously promoting de novo lipogenesis (DNL) and TG accumulation through enhanced translation of mRNAs encoding enzymes involved in fatty acid synthesis. AEG-1 also activates the NF-κB signaling pathway, contributing to hepatic inflammation and fibrosis. In hepatocyte-specific AEG-1 transgenic mice (Alb/AEG-1), inhibition of PPARα and FAO leads to spontaneous NASH development—a condition that is reversed in hepatocyte-specific AEG-1 knockout (AEG-1ΔHEP) mice, suggesting a protective role against diet-induced NASH in the absence of AEG-1 [176]. AEG-1 contains an LXXLL motif at amino acids 21–25 [177], which contributes to its steatotic activity and also regulates its inflammatory and tumorigenic functions, helping maintain a balance in AEG-1’s activity [175]. AEG-1 is also regulated post-translationally via S-palmitoylation at cysteine 75, mediated by the palmitoyltransferase ZDHHC6. This modification negatively regulates AEG-1, restraining its inflammatory and oncogenic functions. Inhibiting depalmitoylases increases AEG-1 palmitoylation, which may suppress both NASH and HCC [178]. In Alb/AEG-1 mice, PPARα inhibition leads to decreased FAO and increased Fas translation, driving DNL and activating NF-κB-mediated inflammation—together contributing to NASH pathology. Therapeutically, hepatocyte-targeted delivery of AEG-1 siRNA via nanoparticles significantly protects wild-type mice from HFD-induced NASH. Thus, AEG-1 inhibition represents a promising therapeutic strategy for NASH patients [176].

### Quaking (QKI) 5

Sirtuins (SIRT1–7) regulate diverse cellular functions through post-translational modification of proteins. Among them, SIRT1 is closely associated with metabolic regulation and has been implicated in the development of NAFLD due to its role in deacetylating various cellular proteins [179]. In mice, downregulation of SIRT1 via small hairpin RNA induces hepatic steatosis, underscoring its protective role in liver metabolism. PPARγ, predominantly expressed in adipose tissue, is upregulated in the livers of NAFLD patients. Increased hepatic PPARγ activity promotes lipid storage and contributes to steatosis [180]. QKI, a member of the signal transduction and activators of RNA (STAR) family of RBPs, is expressed in the liver, with QKI5 being the predominant isoform. Research by Weiyan demonstrated that SIRT1 deacetylates QKI5, thereby influencing TG synthesis in NAFLD mouse models. This regulatory interaction activates the transcription factor Forkhead box protein O1 (FoxO1) through post-transcriptional regulation of PPARα, leading to the inhibition of TG synthesis and slowing NAFLD progression [181].

### Endothelial differentiation-related factor 1 (EDF1)

EDF1, also known as hMBF-1, is a highly conserved intracellular protein composed of 148 amino acids. EDF1 functions as a coactivator for several nuclear receptors involved in lipid metabolism, including liver X receptor alpha (LXRα), steroidogenic factor 1, liver receptor homolog 1, and PPARγ [182, 183]. It facilitates the recruitment of the lncRNA Blnc1, leading to the formation of the LXR ribonucleoprotein transcriptional complex [184]. In cooperation with the LXRα/RXR beta (RXRβ) complex, EDF1 enhances the activity of the SREBP1c promoter, with Blnc1 further amplifying the transcriptional activity of this complex. Overexpression of EDF1 in hepatocytes stimulates the expression of lipogenic genes, and co-expression of Blnc1 intensifies this effect. Additionally, EDF1 is essential for PPARγ transcriptional activation during 3T3-L1 adipocyte differentiation [183], indicating its potential significance in the pathogenesis of NAFLD.

### DEAD-box family

#### DEAD-box protein 1 (DDX1)

DDX1 is a member of the DEAD-box RNA helicase family and is involved in various RNA and DNA processing events, including mRNA translation, miRNA maturation, rRNA processing, tRNA splicing, and the repair of DNA double-strand breaks [185–187]. Studies have shown that DDX1 directly binds to insulin mRNA, and upon stimulation with FFA, DDX1 becomes phosphorylated and dissociates from insulin mRNA, leading to reduced insulin translation [188]. Additionally, DDX1 regulates insulin translation by interacting with eukaryotic initiation factors EIF3a and EIF4B [188]. DDX1 deficiency has been shown to impair calcium influx and insulin secretion in pancreatic β cells [189]. Given its role in insulin metabolism, we hypothesize that DDX1 may influence the development of NAFLD. However, there are currently no direct studies linking DDX1 to NAFLD.

### p68 and p72

p68 and p72, members of the DEAD-box RNA helicase family, are RBPs involved in RNA helicase activity and RNA–protein complex remodeling. They interact with the noncoding RNA Steroid Receptor RNA Activator (SRA), which functions as a transcriptional coactivator for PPARγ and promotes adipocyte differentiation *in vitro*. SRA-deficient mice are resistant to HFD-induced obesity, exhibiting reduced fat mass and increased lean body content. Their livers display fewer lipid droplets, lower expression of lipogenic genes, and reduced hepatic steatosis [190]. In cell models, knockdown of SRA inhibits adipocyte differentiation [191], and SRA has also been shown to promote hepatic steatosis by repressing ATGL expression [192].

### LIN28

LIN28 was originally identified as a regulator of developmental timing in Caenorhabditis elegans [193]. In humans, LIN28 modulates the degradation of let-7 miRNAs and plays roles in various cancers [194, 195]. There are two isoforms of LIN28: LIN28A, which is localized primarily in the cytoplasm, and LIN28B, which is found in both the cytoplasm and nucleus. Unlike LIN28A, LIN28B functions predominantly in the nucleus, where it sequesters primary let-7 transcripts and inhibits their processing [196]. LIN28B was first identified as being overexpressed in HCC [197].

C1632 inhibits LIN28, promoting lipid catabolism and ketogenesis while reducing SREBP1-mediated lipogenesis. These effects collectively limit intracellular lipid accumulation in HepG2 and AML12 cells. In both genetic and dietary mouse models of NAFLD, C1632 activates an anti-steatotic response, suggesting that LIN28 inhibition may offer therapeutic potential for the prevention or treatment of NAFLD [198].

Zhu et al. found that transgenic mice overexpressing LIN28A or LIN28B are resistant to obesity and exhibit improved glucose tolerance. In contrast, muscle-specific Lin28a knockout and let-7 overexpression in mice resulted in glucose intolerance. The regulatory effects of LIN28A/B and let-7 on glucose metabolism are mediated through the insulin–PI3K–mTOR signaling pathway [199]. Additionally, in osteosarcoma cells with high LIN28B expression, aerobic glycolysis was enhanced, while mitochondrial function was impaired [200]. These findings underscore the critical role of LIN28 isoforms in regulating glucose homeostasis, which may influence NAFLD development through their effects on glucose metabolism.

### RPL 8

RCRIN, a conserved read-through circRNA that inhibits metabolic dysfunction-associated steatotic liver disease (MASLD), is downregulated in patients with MASLD. In normal hepatocytes, RCRIN binds to the RPL8 protein and recruits the E3 ubiquitin ligase RNF2 to mediate its degradation. This reduces the number of RPL8-containing ribosomes, thereby inhibiting lipid accumulation and ERS. In MASLD hepatocytes, decreased RCRIN levels lead to the accumulation of RPL8, which contributes to the formation of RPL8-containing ribosomes. This, in turn, enhances lipid accumulation and ERS in the liver. Notably, overexpression of RCRIN or silencing of Rpl8 significantly inhibits the development and progression of MASLD. These findings suggest that RCRIN and RPL8 may serve as valuable biomarkers for MASLD and related metabolic liver disorders. However, whether circulating RCRIN levels in serum could function as a diagnostic marker for MASLD warrants further investigation [201].

### Sarcopenia-related RBPs

Rosenberg first coined the term sarcopenia in 1989 to describe the age-related decline in skeletal muscle mass and volume [202]. Sarcopenia is now recognized as an extrahepatic manifestation of NAFLD [203]. NAFLD and sarcopenia may share common underlying mechanisms, including IR, vitamin D deficiency, chronic inflammation, and reduced physical activity. Sarcopenia has emerged as a novel risk factor for the development of NAFLD [204]. In a prospective observational cohort study, individuals with low muscle mass were found to have a higher risk of developing NAFLD [205]. Those with sarcopenia face a 2.3- to 3.34-fold increased risk of NAFLD [206], and a striking 24-fold increased risk of liver fibrosis [204]. In patients with biopsy-proven NAFLD, sarcopenia has been associated with NASH and significant fibrosis, independent of obesity, inflammation, or IR [207]. Among young and middle-aged populations, both the prevalence and severity of MAFLD are strongly associated with sarcopenia [208]. Evaluating sarcopenia has proven valuable for risk assessment in MAFLD patients [209]. In a prospective study of 225 Caucasian individuals, the prevalence of sarcopenia increased linearly with liver fibrosis severity. Even after adjusting for confounding factors, sarcopenia remained significantly correlated with hepatic steatosis severity [210]. Given the overlapping pathophysiological mechanisms between NAFLD and sarcopenia, it remains unclear whether sarcopenia precedes or follows NAFLD progression [211].

In response to lipopolysaccharide (LPS), TNFα mRNA can be stabilized through phosphorylation of TTP, which promotes TTP binding to the 3′UTR of TNFα mRNA [212]. IL-6 mRNA interacts with multiple RBPs, including HuR, TTP, and AU-rich element-binding protein 1 (AUF1, also known as hnRNP D), suggesting their involvement in the regulation of IL-6 and their potential role in muscle wasting in the elderly [213–215]. QKI protein levels increase during myogenesis and regulate alternative splicing by binding to ACUAA motifs [216]. Depletion of RNA-binding Fox homolog proteins 1 and 2 (RBFOX1 and RBFOX2) in mice leads to severe muscle mass loss and altered splicing of numerous transcripts, highlighting their essential role in muscle maintenance [217]. Masuda et al. [218] reported that in aging skeletal muscle, AUF1 expression increases, TIA-1 and TTP levels decrease, while HuR expression remains unchanged. Polyadenylate-binding nuclear protein 1 (PABPN1) has also emerged as a candidate RBP implicated in muscle aging. Its levels decline in aged muscle, and genetic reduction of PABPN1in mouse models results in muscle atrophy [219, 220]. hnRNP H1 (hnRNPH1) regulates the alternative splicing of RBFOX2 [221], and PABPN1levels have been shown to decrease with age in skeletal muscle [222]. In summary, these RBPs likely contribute to RNA processing and splicing in skeletal muscle and may influence NAFLD development through their role in sarcopenia.

### Interplay across multiple RBPs in NAFLD

Thousands of overlapping binding sites for TTP and HuR have been identified across more than 1300 genes. While TTP promotes mRNA decay, HuR stabilizes and enhances the translation of its target mRNAs. RNA immunoprecipitation (RNA-IP) experiments have shown that TTP can directly bind to and destabilize HuR mRNA. High expression or aberrant nuclear/cytoplasmic distribution of HuR, along with decreased TTP levels, has been observed in various types of cancers [223, 224]. The lncRNA NEAT1, which exists in two major isoforms (NEAT1_v1 and NEAT1_v2), has been implicated in NAFLD. A study by Ahne demonstrated that NEAT1 is oppositely regulated by TTP and HuR in the context of NAFLD [225]. Some RBPs are capable of binding to numerous mRNA targets, potentially producing competitive or synergistic regulatory effects. Such interactions have been explored in other diseases and pathophysiological processes [226–228]. However, studies investigating the combined regulation of specific RNAs by multiple RBPs in the progression or treatment of NAFLD remain limited. Further research is needed to clarify these complex regulatory networks and their therapeutic potential.

Based on the above content, several common and relatively well-studied RBPs implicated in NAFLD are summarized in Table 1. Additionally, Table 2 lists several drugs that can modulate the expression or activity of these RBPs, offering potential therapeutic strategies for the treatment or alleviation of fatty liver disease.

**Table 1:** A comprehensive summary between the RBPs and RBPs-RNA in NAFLD

**RBPs**	**Expression**	**Related RNA**	**Expression**	**Mechanism**	**Results**	**NAFLD**	**Reference**
HuR	*↓*	Acc1, Fas, Elovl6, Fads1 & 2-related genes	*↑*	Fatty acid biosynthesis	Fatty acid biosynthesis	*↑*	[32]
	*↓*	H19	*↑*	miR-130a/PPARγ axis, MLXIPL expression, mTORC1 signaling	Steatosis and lipid accumulation	*↑*	[35, 36]
	*↑*	PTEN mRNA	*↑*	increases stability and translation of PTEN	Lipid depositionin hepatocytes*↓*	*↓*	[30]
	*↓*	Apob pre-mRNA, UQCRB, NDUFB6 mRNA	*↓*	Regulate splicing of Apob mRNA and translation of UQCRB and NDUFB6	Liver lipid transport and ATP synthesis*↓*	*↑*	[31, 40]
	*↓*	ADH1C, CYP4A1 1, ALDH5A 1	*↓*	LINC01 018 binds to HuR and regulate its activity	Regulates hepatic fatty acid metabolism	*↑*	[41]
	*↑*	APOA4 mRNA	*↑*	HuR–A POA4-AS complex stabilizes APOA4 mRNA	Increases TGs and TC	*↑*	[43, 43]
	*↑*	C/EBPβ mRNA	*↑*	Binds to 3’UTR of C/EBP βmRNA and increases its stability and translation	Aggravates hepatic PPARγ activation, NF-κB activation	*↑*	[44–46]
	*↑*	HAMP mRNA	*↑*	Binds to the 3’UTR of HAMP mRNA, up-regulate HAMP mRNA in hepatocytes	Lipid content*↑*	*↑*	[48]
	*↑*	LDLR mRNA	*↑*	Stabilizes LDLR mRNA, increase LDLR in the liver	LDLC in the plasma *↓*	*↓*	[49]
	*↑*	ABCA1 mRNA	*↑*	Promotes ABCA1 through 3’UTR binding-mediated mRNA stabilization	Cholesterol efflux, HDLC biogenesis	*↓*	[50]
	*↑*	ATGL mRNA	*↑*	Increases its stability and protein level of ATGL mRNA	TC and HDLC*↓*	*↓*	[29]
	*↓*	MnSOD and HO-1 mRNA	*↓*	Protective role of HuR against oxidative stress *↓*	Oxidative stress *↑*	*↑*	[56]
HnRNPI (PTBP1)	*↑*	H19, SREBP1	*↑*	H19/PTBP1/SREBP1 forward amplifying pathway	Lipogenic program	*↑*	[77]
		Sc5d, Lss, Fdps, and Hmgcs1	*↑*	hLMR1 enhances the binding of PTBP1 on the promoters of Sc5d, Lss, Fdps, and Hmgcs1	Cholesterol synthesis, adipogenic differentiation	*↑*	[79]
hnRNPU	*↑*	lncRHL	*↑*	lncRHL/hnRNPU/BMAL1/MTTP axis	VLDL secretion in hepatocytes	*↓*	[83]
hnRNPA1	*↓*	gys1 mRNA	*↓*	gys1 mRNA stability*↓*	Glycogen storage *↓* IR*↑*	*↑*	[91]
	*↑*	lncRNA SHGL	*↑*	Activates PI3K/Akt pathway activate CaM/Akt pathwa y repress mTOR/SREBP-1C pathway	Hyperglycemia and steatosis*↓*	*↓*	[92, 95]
		H19	*↑*	Increases translation of PGC1a and CPT1b	Lipidectopic deposition and IR*↓*	*↓*	[93]
hnRNPC	*↓*	circ-Tulp4	*↑*	Inhibits the HNRNPC/ABHD6 axis	Reduces apoptosis	*↓*	[98]
p62	*↑*	miR-130b-5p	*↓*	AKT pathway	Expression of lipid synthesis genes*↓*	*↓*	[116]
TTP	*↓*	FGF21 mRNA	*↑*	FGF21*↑*	Improves glucose tolerance and insulin sensitivity	*↓*	[129]
CPEB1	*↓*	miR-122	*↓*	CircRNA-002581-miR-122-CPEB1 axis PTEN-AMPK-mTOR pathway	Autophagy *↑*	*↓*	[134–136]
TDP-43	*↑*	lncLSTR	*↑*	TDP-43/FXR/apoC2 pathway	TG*↓*	*↓*	[142]
YAP	*↑*	lncARSR	*↑*	LncARSR activates IRS2/AKT pathway by reducing YAP1 phosphorylation	Lipid accumulation	*↑*	[143]
		MAYA	*↓*	Regulates iron overload in hepatocytes	Cellular senescence*↓*	*↓*	[149, 150]
	*↓*	SRD5A3AS1	*↑*	SRD5A3-AS1 inhibite miR-1205, upregulating NF2, upregulate NF2 negatively regulate YAP1	NAFLD cell proliferation*↓*, IL-6, TGF-β1, α-SMA *↓*	*↓*	[155]
YBX1	*↑*	ULK1 mRNA	*↑*	YBX1 enhances ULK1- and ULK2-mediated autophagy	Adipogenesis	*↑*	[167]
AEG-1	*↑*	Fatty acid synthesis related mRNA	*↑*	Fatty acid synthesis*↑*	DNL and TG*↑*	*↑*	[176]
EDF1	*↑*	Blnc1 RNA	*↑*	Facilitates formation of LXR ribonucleoprotein transcriptional complex	Stimulates lipogenic gene expression	*↑*	[183, 184]
p68 and p72	*↑*	SRA	*↑*	Adipocyte differentiation repress adipose triglyceride lipase	Lipogenesis	*↑*	[190–192]
RPL 8	*↑*	RCRIN	*↓*	Form of RPL8-containing ribosomes	Lipid accumulation and ERS	*↑*	[201]

**Expression:** The increase or decrease in the expression levels of RBPs and related RNAs. **Results:** The impact of RBPs on NAFLD and its metabolism through relevant mechanisms. HuR: Human antigen R; NAFLD: Nonalcoholic fatty liver disease; TG: Triglyceride; TC: Total cholesterol; ATGL: Adipose triglyceride TG lipase; C/EBPβ: CCAAT/enhancer-binding protein beta; PPARγ: Proliferator-activated receptor gamma; NF-κB: Nuclear factor kappa B; FXR: Farnesoid X receptor; APOA4: Apolipoprotein A-IV; MnSOD: Manganese-dependent superoxide dismutase; HO-1: Heme oxygenase-1; mTORC1: Mechanistic target of rapamycin complex 1; Acc1: Acetyl-CoA carboxylase; Fas: Fatty acid synthase; Elovl6: Elongation of very-long-chain fatty acids member 6; Fads1&2: Fatty acid desaturases 1 and 2; SphK2: Sphingosine kinase 2; ERS: Endoplasmic reticulum stress; TTP: Tristetraprolin; RBP: RNA-binding protein; YAP: Yes-associated protein 1; AEG-1: Astrocyte elevated gene-1; HMGCS1: Hydroxymethylglutaryl-CoA synthase 1; MLXIPL: MLX-interacting protein-like; 3’ UTR: 3′ untranslated region; SREBP1c: Sterol regulatory element-binding protein 1c; hLMR1: Human lncRNA metabolic regulator 1; PTBP1: Polypyrimidine tract-binding protein 1; MTTP: Microsomal triglyceride TG transfer protein; VLDL: Very-low-density lipoprotein; gys1: Glycogen synthase 1; PI3K: Phosphatidylinositol 3-kinase; mTOR: Mammalian target of rapamycin; hnRNPA1: Heterogeneous nuclear ribonucleoprotein A1; CaM: Calmodulin; FGF21: Fibroblast growth factor 21; CPEB1: Cytoplasmic polyadenylation element-binding protein 1; TDP-43: Tat-activating regulatory DNA-binding protein 43; IRS2: Insulin receptor substrate 2; YXB1: Y-box binding protein-1; ULK1: Unc-51–like kinases 1; ULK2: Unc-51–like kinases 2; EDF1: Endothelial differentiation-related factor 1; Blnc1: Brown fat lncRNA 1; SRA: Steroid receptor RNA activator; GDNL: De novo lipogenesis; CPT1b: Carnitine palmitoyltransferase 1B; PGC1α: PPARγ coactivator 1-alpha.

**Table 2:** Potential drugs targeting RBPs and RBPs-RNA in NAFLD

**RBPs**	**Drugs**	**Diseases**	**Function**	**Stage**	**Reference**
HuR	TUDCA	NAFLD	Inhibites ERS	Animal experiment	[28]
HuR	Insulin sensitizers	NAFLD	Ameliorates insulin resistance	Speculation	[40]
HuR	OCA	NAFLD	Inducts hepatic HuR expression	Animal experiment	[49]
HuR	SAM	NAFLD	Maintains HuR methylation	Animal experiment	[61]
HuR	Flavonoids	NAFLD	Inhibites HuR and reduce expression of HuR target genes	Speculation	[62]
TTP	Metformin	NASH	Decreases TNF-α production in KCs	Animal experiment	[125]
YAP	Si-Ni-San	NAFLD	Reduces YAP expression	Cell and animal experiment	[152]
YAP	RA	NAFLD	Down-regulates the expression of YAP	Cell and animal experiment	[153]
YAP	Curcumol	NAFLD	Inhibites hepatocyte senescence	Cell and animal experiment	[154]
YAP	SRD5A3-AS1	NAFLD	Negatively regulates YAP	Animal experiment	[155]
YAP	LMY	NAFLD	Suppresses YAP1-mediated Foxm1 activation	Animal experiment	[156]
YAP	Magnesium isoglycyrrhizinate	HSC	Inhibites Hippo/YAP signaling pathway	Cell and animal experiment	[161]
YAP	HMGCS1	NAFLD related cancer	Activates YAP	Cell and animal experiment	[162]
EIF4E	Rapamycin	NAFLD	Inhibites phosphorylation of EIF4E	Cell and animal experiment	[171–173]
AEG-1	ZDHHC6	NASH	Increases AEG-1 palmitoylation	Animal experiment	[178]
Lin28	C1632	NAFLD	Inhibites Lin28	Cell and animal experiment	[198]

TUDCA: Tauroursodeoxycholic acid; NAFLD: Nonalcoholic fatty liver disease; SAM: S-adenosylmethionine; NASH: Nonalcoholic steatohepatitis; TTP: Tristetraprolin; ERS: Endoplasmic reticulum stress; HuR: Human antigen R; RBP: RNA-binding protein; OCA: Obeticholic acid; YAP: Yes-associated protein 1; AEG-1: Astrocyte elevated gene-1; EIF4E: Eukaryotic initiation factor 4E; LMY: Lian-Mei-Yin; HMGCS1: Hydroxymethylglutaryl-CoA synthase 1; RA: Rosmarinic acid; HSC: Hepatic stellate cell; KC: Kupffer cell.

## Conclusion

Despite the growing body of literature on RBPs and their roles in NAFLD, existing research remains fragmented and lacks comprehensive, systematic reviews. Given the high prevalence and clinical significance of NAFLD, a thorough understanding of the post-transcriptional regulatory functions of RBPs is essential for the development of innovative RNA-based therapies. This review aims to clarify the pathogenesis of NAFLD by highlighting recent advances in our understanding of the involvement of various RBPs in disease development. Ultimately, we anticipate that a more complete picture of the dynamic, RBP-mediated regulatory network in NAFLD will emerge. Correcting gene expression abnormalities through targeted modulation of RBPs holds promise as an effective therapeutic strategy. RNA-based therapies that mimic protective RBPs or inhibit pathogenic ones could provide new avenues for intervention. However, improving target specificity remains a major challenge that must be addressed. Moreover, while direct links between specific RBPs and NAFLD are still being established, most existing data derive from *in vivo* and *in vitro* models. Clinical studies in patients are urgently needed to validate these findings and assess the utility of diagnostic or therapeutic panels based on the RBPs discussed in this review. Despite the many unresolved questions surrounding RBPs in NAFLD, the current body of knowledge and accumulating evidence suggest that RBPs may open a new era in the treatment of fatty liver disease.

Future research in this field could evolve along several technological fronts, including genomics, high-throughput screening (HTS), and gene editing, to deepen our understanding and expand applications related to RBPs in NAFLD. The widespread adoption of HTS methods, such as RNA sequencing (RNA-seq), will facilitate the identification of novel RBPs and potential therapeutic targets, accelerating the discovery of candidate molecules for NAFLD treatment and shortening the drug development timeline. Gene editing technologies, particularly CRISPR-Cas9, will aid in the creation of precise NAFLD animal models, offering new avenues for advancing precision medicine. As these technologies continue to develop, drug development costs are expected to decrease significantly. Moreover, RBP-targeted therapies may exhibit more precise mechanisms of action, potentially reducing side effects and adverse reactions. This could enhance therapeutic outcomes while lowering the economic burden on patients.
